# Controversial Surgical Approach to Recurrent Gallstone Ileus

**DOI:** 10.7759/cureus.66893

**Published:** 2024-08-14

**Authors:** Victor H Nuño-Rodriguez, Nora L Flores-Olmos, Jaime A Alvarez-Gutierrez

**Affiliations:** 1 General Surgery, Regional Hospital “Dr. Valentin Gomez Farias”, Institute of Security and Social Services for the State Workers, Zapopan, MEX

**Keywords:** pneumobilia, enterolithotomy, bilio-enteric fistula, cholelithiasis, biliary stone, gallstone ileus

## Abstract

Gallstone ileus is a rare but potentially serious complication of gallstone disease, which presents as a mechanical intestinal obstruction due to impaction and fistulization of a gallstone, most commonly in the small intestine. Since it usually occurs in elderly patients, the symptoms can be very diverse and with a late presentation.

We present the case of a 90-year-old patient with intestinal obstruction and acute abdominal pain who experienced gallstone ileus and underwent surgery, and a few days after being discharged returned with a recurrence of the symptoms, was re-operated, and a second stone was found.

## Introduction

Gallstone ileus is defined as a mechanical obstruction of the small intestine induced by an impacted biliary stone [1,2] that, due to inflammatory changes, pressure erosion, and gallbladder ischemia, causes a bilio-enteric fistula [3,4]. It has a high mortality rate of between 12%-27% [5]. The most common site of stone impaction is the ileum, in 50%-65% of cases [1,6].

Gallstone ileus occurs in 0.15%-1.5% of cases with cholelithiasis and has a recurrence of 5%-8% [1,7].

It does not have distinctive symptoms, which results in a late diagnosis. It may be preceded by a diagnosis of cholelithiasis, episodes of biliary colic, and data suggestive of intestinal obstruction such as abdominal pain (91.5%), nausea and vomiting(87%), and abdominal distention (84%) [1,3].

There are three surgical approaches for gallstone ileus. The first consists of an enterolithotomy to manage intestinal obstruction (mortality 4.2%). The second corresponds to performing the procedure in two stages: first, the surgical extraction of the impacted stone, and second, the repair of the fistula [1]. The third corresponds to an enterolithotomy and repair of the biliary fistula as a one-stage procedure; however, it has been seen that this treatment causes a higher mortality (22%) [5]. However, the simple enterolithotomy and the two-stage surgery may present gallstone ileus recurrence, cholangitis, and a higher risk of cancer development due to remaining cholecysto-intestinal fistula [1].

## Case presentation

**Figure 1:**
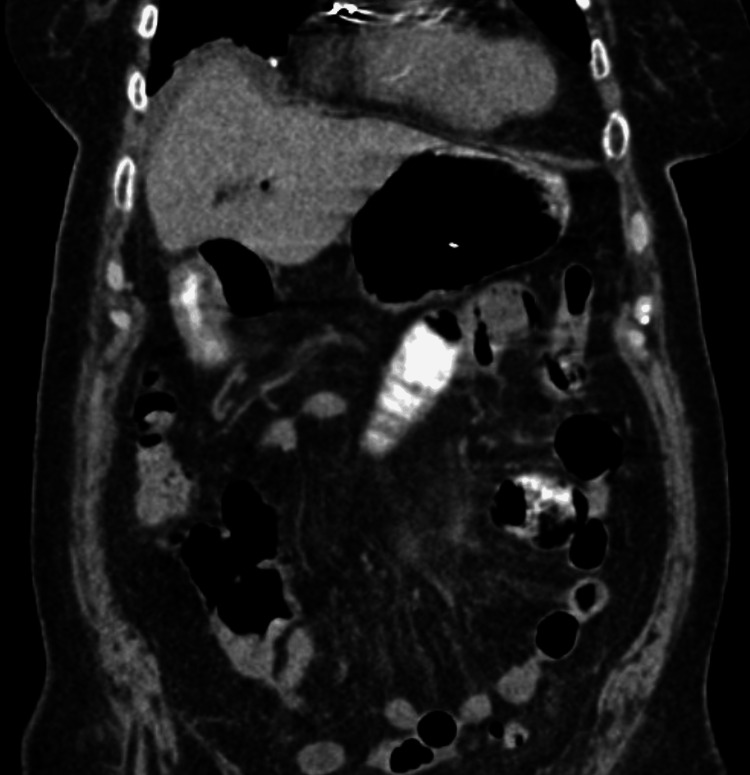
Computed tomography showing pneumobilia.

We present the case of a 90-year-old woman, hypertensive, with a pacemaker and a history of hysterectomy 52 years ago, open appendectomy 40 years ago, knee prosthesis with subsequent removal and fixation six years ago, and cardiac catheterization three months previously. Upon questioning, she reported 72 hours of evolution of generalized abdominal pain accompanied by nausea as well as constipation. Upon admission to the emergency room, she presented with intense abdominal pain, conservative management was given due to intestinal sub-occlusion but due to the worsening of the condition, it was decided an evaluation by general surgery, finding the patient restless, pale, with peristalsis abolished, intense pain predominantly in mesogastrium. Blood studies revealed leukocytosis with neutrophilia. Abdominal radiography with data of intestinal obstruction and tomography showed pneumobilia without evidence of stone or site of occlusion (Figure 1).

The patient underwent an exploratory laparotomy, finding a subhepatic plastron with firm adhesions. During the systematic exploration of the entire intestine, a firm intraluminal body was palpated at 55 cm from the angle of Treitz, which was manually mobilized distally, advancing it to 170 cm from the angle of Treitz.

**Figure 2:**
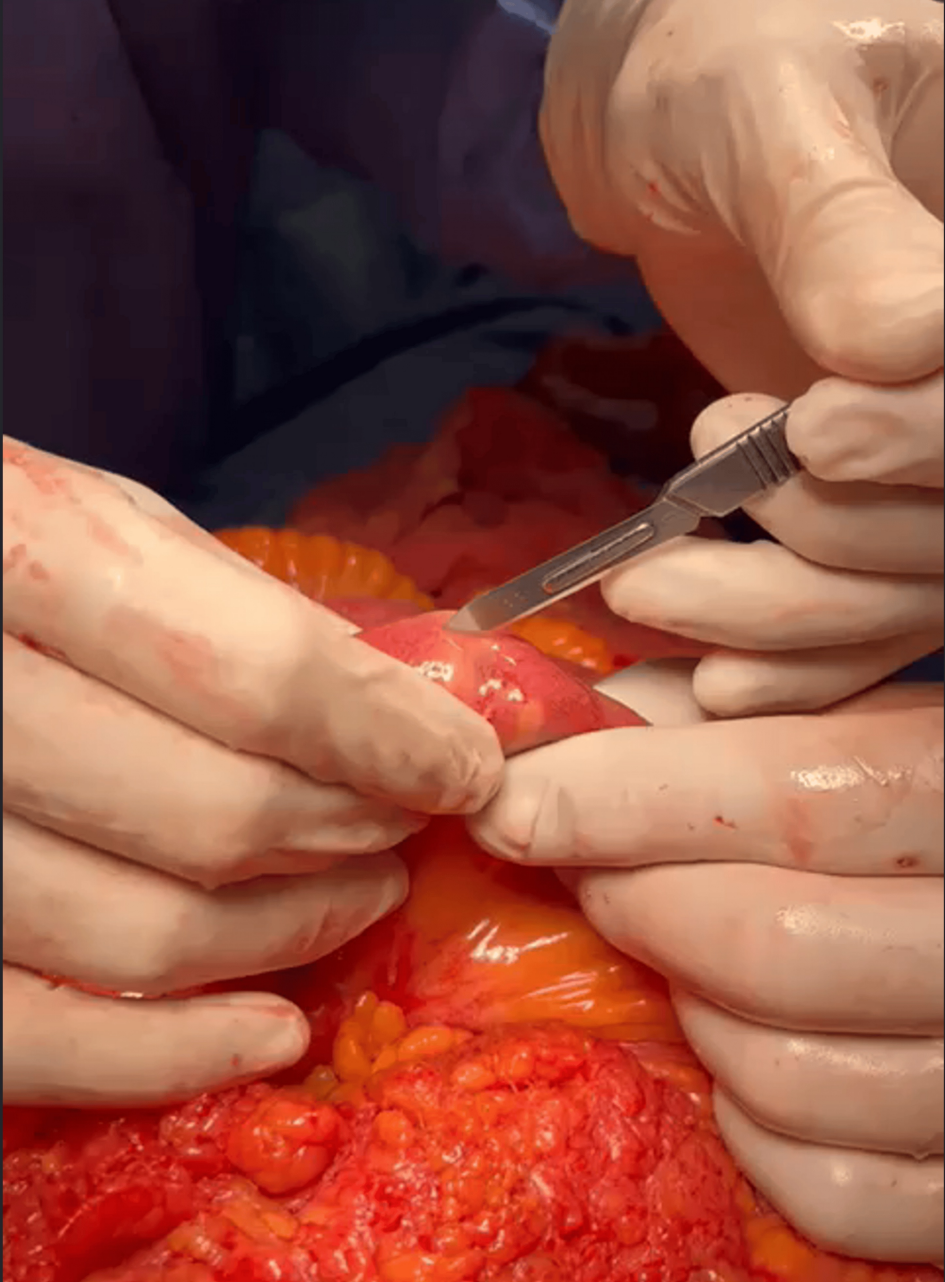
Identification of intraluminal body and preparing for enterotomy

**Figure 3:**
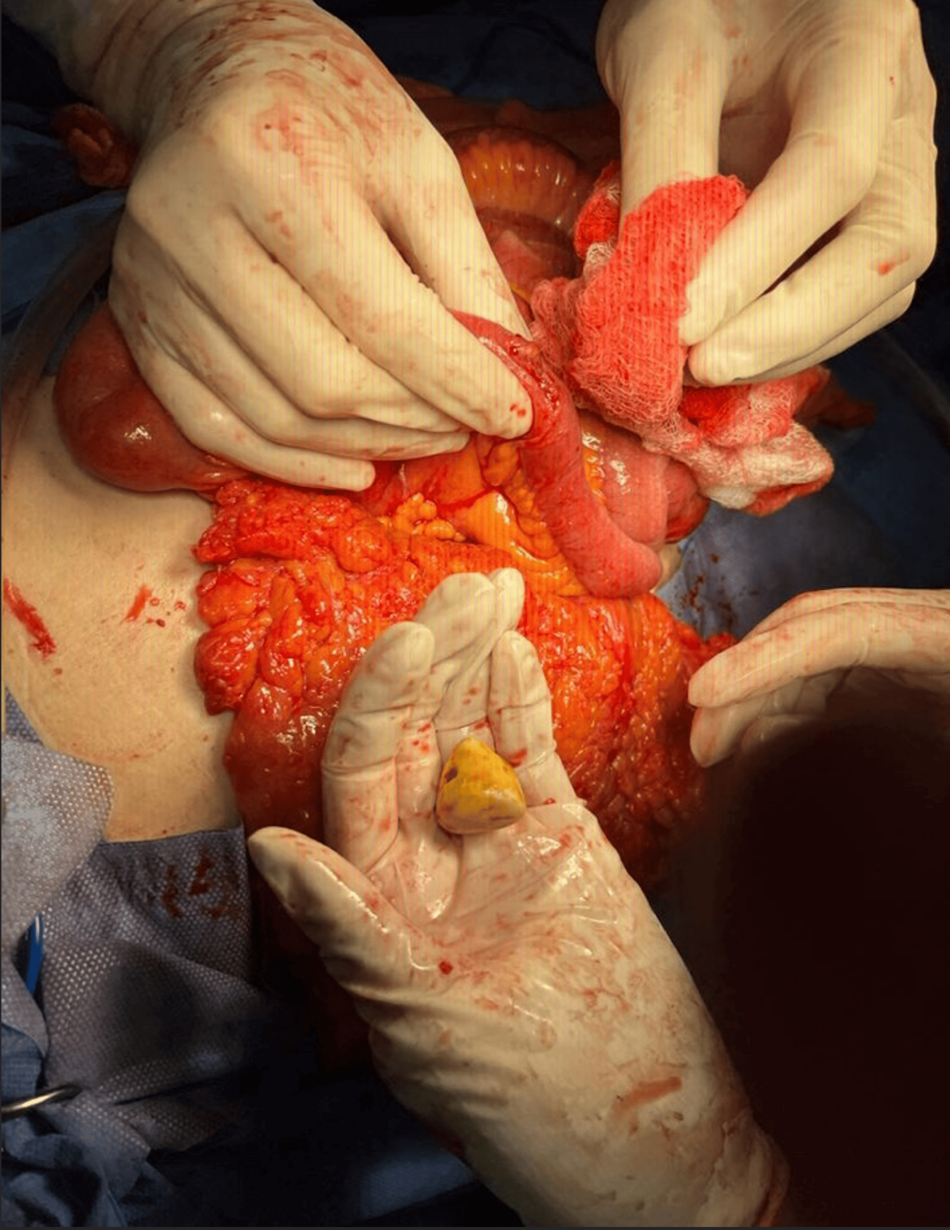
Extraction of biliary stone

A longitudinal enterotomy was performed (Figure 2), finding a 2 x 2.5 cm biliary stone (Figure 3). The enterotomy was closed in two layers and in a transverse direction. The abdominal cavity was cleaned and drains were placed.

**Figure 4:**
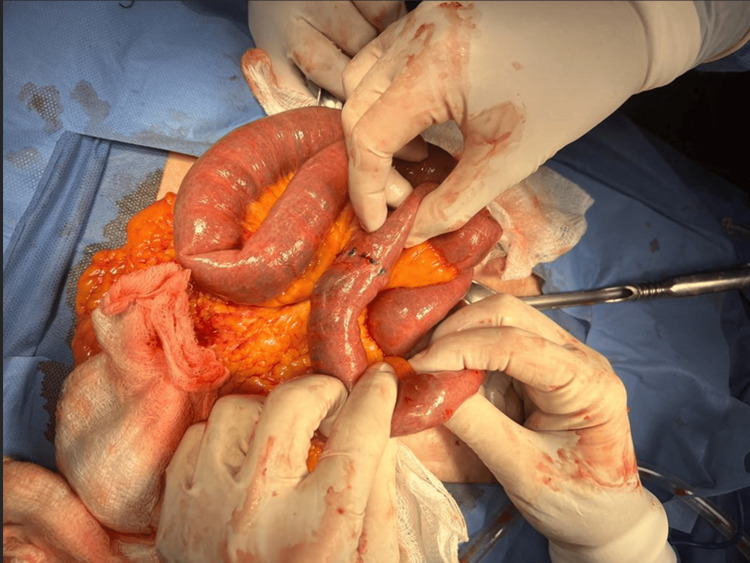
Second stone found close to the previous enterotomy

The postoperative period was long but uneventful, and the patient was discharged on the sixth day. However, 24 hours after discharge, she returned to the emergency department with data suggestive of an acute abdomen accompanied by vomiting coffee grounds. Due to the history, a new exploratory laparotomy was decided. A cavity exploration was performed, finding a new impacted biliary stone 10 cm proximal to the previous enterotomy (Figure 4). A new enterotomy was performed, the stone was extracted, and no evidence of more stones was found.

The patient remains hospitalized for post-surgical monitoring and, due to good clinical progress, she was discharged to her home.

## Discussion

Recurrent gallstone ileus is a rare but important complication of gallstones, which can occur even after cholecystectomy [8]. Risk factors for the development of gallstone ileus include the presence of large gallstones and abnormal anatomy of the biliary tract [1].

The treatment of gallstone ileus is a bit controversial since ideally, it would require the extraction of the stone from the intestine as well as cholecystectomy and fistula repair; however, the morbidity and mortality associated with this procedure are significant considering the age of presentation of gallstone ileus as well as the comorbidities of the affected patients. So the alternatives are to perform the procedure in two stages: only the surgical extraction of the impacted stone in the first stage, and in the second surgical stage, the repair of the fistula [1] or the enterolithotomy alone with subsequent monitoring, waiting for spontaneous closure of the fistula [9].

In our patient, due to the admission conditions, age, and history, during the first intervention, due to the emergency, we decided not to manipulate the plastered area, and after reviewing the entire intestine and ensuring that there was only one stone, we resolved the occlusion by performing longitudinal enterolithotomy at the antimesenteric border. To reduce the risk of stenosis, a transverse closure was performed, suturing in two planes to prevent leakage. The integrity of the closure was checked in the second procedure.

During the second procedure, due to the severe inflammatory process and the adhesions around the gallbladder, and the quality of the surrounding tissues, it was again decided to perform enterolithotomy alone to avoid iatrogenic injuries. Upon discharge, after consulting with the patient and caretakers, considering the risk-to-benefit ratio, it was decided to continue follow-up by consultation without considering a second-stage procedure.

## Conclusions

Recurrent gallstone ileus is a rare but potentially serious complication of gallstones. In our surgical team, taking into account all the comorbidities presented by our patient, as well as the general condition, we have opted to perform enterolithotomy alone in order to reduce surgical time and promote the stabilization of the complicated patient.

It is important to note that during the procedure a complete intestinal exploration should be ensured in case more than one stone is found to avoid increasing morbidity. In the context of the uncomplicated patient, without comorbidities and in good general condition, performing the one-stage procedure could be appropriate.
